# Supplementary material for: Identification of photoperiod-induced specific miRNAs in the adrenal glands of Sunite sheep (Ovis aries)
Source: Front Vet Sci. 2022 Jul 22;9:888207. doi: 10.3389/fvets.2022.888207 (PMC9354845; doi:10.3389/fvets.2022.888207)
Supplement: Supplementary file 1 [file Data_Sheet_1.ZIP › Supplementary file/miRDeep2 file.zip!/miRDeep2 file/Know_Structure/LP42A3/oar-mir-136.pdf]

miRBase precursor : oar-mir-136
Total read count : 14475
oar-miR-136 read count : 4479
remaining reads : 9996

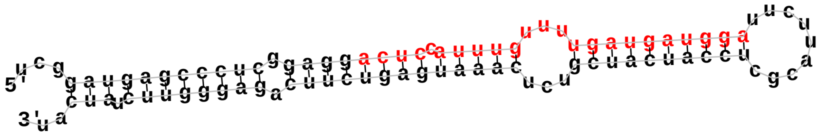

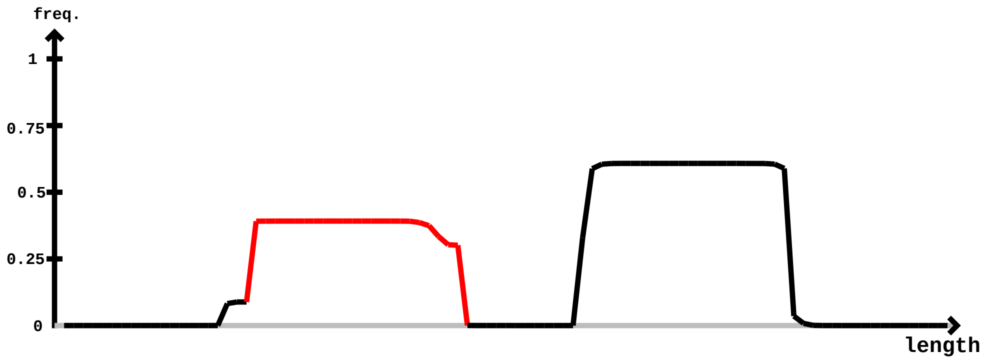

oar-miR-136

5'- ucggaugagcccucggaggacuccauuuguuuugaugauggauucuuacgcuccaucaucgucucaaaugagucuucagaggguucuaucau -3'	exp		
...(((((((((((.((((((((.(((((...((((((((((.........))))))))))...))))))))))))).)))))))).)))..	reads	mm	sample
ucggaugagcccucggagg...	1	0	seq
.cggaugagcccucggagg...	1	0	seq
................aggacuccauuuguuuug..	1	0	seq
................aggacuccauuuguuuuga...	8	0	seq
................aggacuccauuuguuuugau..	68	0	seq
................aggacuccauuuguuuugaug...	170	0	seq
................aggacuccauuuguuuugauga..	571	0	seq
................aggacuccauuuguuuugaugau...	373	0	seq
................aggacuccauuuguuuugaugaug..	1	0	seq
.................ggacuccauuuguuuugau..	2	0	seq
.................ggacuccauuuguuuugauga..	16	0	seq
.................ggacuccauuuguuuugaugau...	65	0	seq
.................ggacuccauuuguuuugaugaug..	2	0	seq
...................acuccauuuguuuugau..	1	0	seq
...................acuccauuuguuuugaugau...	8	0	seq
...................Ccuccauuuguuuugaugaug..	2	1	seq
...................acuccauuuguuuugaugaug..	22	0	seq
...................acuccauuuguuuugaugaugg...	4349	0	seq
...................acuccauuuguuuugaugaugga..	5	0	seq
....................cuccauuuguuuugaugaug..	1	0	seq
....................cuccauuuguuuugaugaugg...	2	0	seq
.....................uccauuuguuuugaugaugA...	1	1	seq
.....................uccauuuguuuugaugaugg...	2	0	seq
.........................uuuguuuugaugaugAauucu..	1	1	seq
....caucaucgucucaaaug......................	2	0	seq
....cCucaucgucucaaaug......................	1	1	seq
....caucaucgucucaaauga.....................	1	0	seq
....caucaucgucucaaaugag....................	1	0	seq
....caucaucgucucaaaugagu...................	32	0	seq
....caucaucgucucaaaugaguc..................	181	0	seq
....caucaucgucucaaaugagucu.................	4513	0	seq
....caucaucgucucaaaugagucuu................	37	0	seq
....aucaucgucucaaaugagu...................	3	0	seq
....aucaucgucucaaaugaguc..................	50	0	seq
....aGcaucgucucaaaugagucu.................	1	1	seq

oar-miR-136

```
ucggaugagcccucggaggacuccauuuguuuugaugauggauucuuacgcuccaucaucgucucaaaugagucuucagaggguucuaucau
......................................................aucaucgucucaaaugagucu.................   3445   0   seq
......................................................aucaucgucucaaaugagucuu................   243    0   seq
......................................................aucaucgucucaaaugagucuuc...............   7      0   seq
.......................................................ucaucgucucaaaugaguc..................   1      0   seq
.......................................................ucaucgucucaaaugagucu.................   47     0   seq
.......................................................ucauUgucucaaaugagucu.................   4      1   seq
.......................................................ucaucgucucaaaugagucuu................   112    0   seq
.......................................................ucaucgucucaaaugagucuuc...............   71     0   seq
.......................................................ucaucgucucaaaugagucuuca..............   4      0   seq
.......................................................ucaucgucucaaaugagucuucaga............   1      0   seq
........................................................caucgucucaaaugagucuu................   3      0   seq
........................................................caucgucucaaaugagucuuc...............   30     0   seq
........................................................caucgucucaaaugagucuuca..............   6      0   seq
.........................................................aucgucucaaaugagucu.................   4      0   seq
.........................................................aucgucucaaaugagucuu................   1      0   seq
.........................................................aucgucucaaaugagucuuca..............   1      0   seq
```
